# “My Child has Cerebral Palsy”: Parental Involvement and Children’s School Engagement

**DOI:** 10.3389/fpsyg.2016.01765

**Published:** 2016-11-11

**Authors:** Armanda Pereira, Tânia Moreira, Sílvia Lopes, Ana R. Nunes, Paula Magalhães, Sonia Fuentes, Natalia Reoyo, José C. Núñez, Pedro Rosário

**Affiliations:** ^1^Department of Applied Psychology, School of Psychology, University of MinhoBraga, Portugal; ^2^Facultad de Educación, Universidad Central de ChileSantiago, Chile; ^3^Departamento de Psicología, Universidad de ValladolidValladolid, Spain; ^4^Department of Psychology, Universidad de OviedoOviedo, Spain

**Keywords:** school engagement, cerebral palsy, semi-structured interview, parental style, autonomy, thematic analysis

## Abstract

Engaged students tend to show school-committed behaviors (e.g., attend classes, get involved with the learning process), high achievement, and sense of belonging. However, students with disabilities are prone to show a lack of engagement with school due to the specific difficulties they have to handle. In fact, children with disabilities are likely to show poor participation in school when compared with children without disabilities. This poor involvement is related to their low autonomy to participate in the school activities, which, in turn, results in low school engagement. Parents play a crucial role in their children’s education. Parental involvement in school activities promotes autonomous behaviors and, consequently, school engagement. In fact, extant literature has shown close relationships between parental involvement, school engagement, and academic performance. Yet, parental involvement in school activities of children with Cerebral Palsy (CP) has received little direct attention from researchers. These children tend to display lower participation due to the motor, or cognitive, impairments that compromise their autonomy, and have a high likelihood to develop learning disabilities, with special incidences in reading and arithmetic. Therefore, our aim is twofold, to understand the parental styles; and how the perceived parental involvement in school activities is related to their children school engagement. Hence, 19 interviews were conducted with one of the parents of 19 children with CP. These interviews explored the school routines of children and the perceived involvement of parents in those routines. Additionally, children filled out a questionnaire on school engagement. Results show that the majority of the parents were clustered in the Autonomy Allowance and Acceptance and Support parental style, and the majority of their children were perceived as autonomous. Moreover, about a half of the children reported a high level of school engagement. Finally, neither children’s autonomous behaviors reported by parents, nor parental style, seem to be related with the children’s level of school engagement. Rehabilitation centers and schools could consider training parents/caregivers focusing on their educational needs, promotion of reflections on the usefulness of applying autonomy promotion strategies with their child, and foster their involvement.

## Introduction

### School Engagement

The concept of school engagement (SE) emerges as closely related to educators’ increasing concern about the high rates of school dropout and low academic achievement (Finn, 1993; Finn and Rock, 1997; European Commission, 2014). SE is a multidimensional and multifaceted construct involving three interrelated dimensions: students’ behaviors, emotions, and cognition (Fredricks et al., 2004; Wang and Holcombe, 2010). Specifically, behavioral engagement can be conceptualized in three levels: (i) school attendance and fulfillment of schoolwork, (ii) participation in class, and (iii) active participation, such as doing extra work for school courses (Finn and Rock, 1997). Emotional engagement is related to feelings about school. Identification with school is crucial for the involvement in activities and is closely related to students’ feelings of belongingness (Connell et al., 1994). Lastly, cognitive engagement comprises efforts, will and deliberation, to master complex skills, and is closely related to self-regulated strategies (Fredricks et al., 2004; Rodríguez et al., 2014; Rosário et al., 2015). Together, these three components of engagement can enhance educational performance (Finn and Zimmer, 2012).

#### School Engagement in Children with Disabilities

Children with disabilities struggle with difficulties in school, being, as a consequence, prone to develop a poor SE (Blackorby and Cameto, 2004). This educational scenario may be related to their high levels of school absenteeism. In fact, children with disabilities miss, on average, 3 weeks of school in a school year (Blackorby and Cameto, 2004). Contrary to students without disabilities, in which absenteeism may be associated with “skipping school,” students with disabilities frequently involuntarily miss classes due to health issues. School absenteeism constitutes, therefore, a real barrier to the progression in the learning processes and in school participation. Regarding school participation, Finn (1993) considered that the way students identify and involve themselves with the school environment reflects how they are engaged with school. Specifically, the time children spend interacting in social and physical environments (e.g., with peers, materials) are associated with SE (Maher Ridley et al., 2000). Hence, the level and quality of participation in school activities play an important role in developing SE in students with disabilities (Almqvist et al., 2006). In fact, children without disabilities show a higher rate of participation in autonomous activities than children with disabilities (Eriksson and Granlund, 2004).

Participation, as defined by the International Classification of Functioning, Health, and Disability, is the active involvement of individuals in their life situations considering their social, functional, and health dimensions (World Health Organisation [WHO], 2001). In addition, according to Eriksson and Granlund (2004), participation demands that students experience feelings of belonging, as well as their perceived control and involvement with the school context, which will prevent feelings of school alienation. This school alienation is characterized by feelings of estrangement and social isolation, which may trigger dropout or school failure (Finn, 1993; Fredricks et al., 2004). Children’s active participation requires, therefore, not only their personal will, but also their capacity to autonomously assume the control of their participation in the school activities (Almqvist et al., 2006). Yet, students with disabilities face difficulties in controlling activities on their own due to their lower level of autonomy in comparison with students without disabilities (Blackorby and Cameto, 2004). Autonomous behavior requires self-initiation and self-regulation competences (Wang and Holcombe, 2010), and children with physical or cognitive disabilities are likely to show limitations in their autonomy. Additionally, regarding the physical disabilities, the degree of body limitation has more influence in the children’s participation than the type of body disability [i.e., the area of the body limitation have more influence in the children’s autonomy if the limitation is classified as severe (e.g., IV or V in GMFCS levels)] (Simeonsson et al., 2001). Lastly, the school environment plays an important role on the limited autonomy of children with disability. The spatial organization of the school environment, for example, informs children about their possibilities to move and participate in the classroom routines (e.g., if do not have space to drive my wheelchair in class, I’ll not go to the blackboard check my homework). Those perceptions, consequently, influence children expectations to participate in the activities developed in that context (Eriksson and Granlund, 2004).

However, the difficulties inherent to the disabilities, and the physical constraints of the environment, are not the only explanations to the limited autonomy of children with disabilities. In fact, parents of these children, and society, are likely to create low expectations about the autonomy of children with disabilities and act accordingly, for example by controlling their behavior. These low expectations may contribute to strengthen these children’s sense of low autonomy or lack of participation in school activities (Blackorby and Cameto, 2004; Elad et al., 2013).

### A Specific Disability: Cerebral Palsy

Among the childhood physical disorders, Cerebral Palsy (CP) is considered the most common, with a lifelong impact (Rosenbaum, 2003; Aisen et al., 2011; Novak et al., 2013). In fact, it is estimated that the prevalence of this clinical condition is about 3 to 4 children per 1000 live births (Yeargin-Allsopp et al., 2008) and is present in the lives of about 17 million people (Cerebral Palsy Alliance [CPA], 2013). CP subsumes a group of neurological, non-progressive, and permanent (but changeable) disorders that mainly affect movement and posture. This disorder can result from lesions or disturbances in early brain development during the prenatal, perinatal, or postnatal periods (Rosenbaum, 2003; Bax et al., 2005; Rosenbaum et al., 2007).

The classification of CP depends on a neurological examination to evaluate the nature of the motor impairment and the topographic type, i.e., parts of the body affected (Bax et al., 2005; Aisen et al., 2011). The nature of motor impairment includes the following classifications: dyskinetic, ataxic, spastic, and mixed. Dyskinetic is characterized by uncontrolled, writhing, and slowed movements, and, sometimes, drool and grimace. Ataxic is the rarest form of CP and is characterized by difficulties in coordination and balance, expressed in gait and fine motor problems. Spastic is the most common and is characterized by the presence of deep tendon reflexes and muscle tone, tremors, muscle weakness, and gait problems. Frequently, spastic is related to dysarthria, oromotor problems with drooling and swallowing difficulties. Lastly, a mixed clinical picture can also be observed and represents 30% of the cases of children with CP (Sankar and Mundkur, 2005; Straub and Obrzut, 2009; Aisen et al., 2011; Richardson et al., 2014). In addition, the topographic classification adds information about the part of the body impaired. It includes diplegia (lower limbs more affected than upper limbs), hemiplegia (upper and lower unilateral extremity impairment), and quadriplegia (severe four-extremity impairment). The severity of these clinical pictures can be very diverse, with distinct repercussions in the autonomy of the individual. According to the Gross Motor Function Classification System (GMFCS; Palisano et al., 1997; Andrada et al., 2007), the movement ability (e.g., self-initiated movement) can be evaluated in a range of five levels of functionality (I, minor difficulties to V, major difficulties). Therefore, all these classifications are important to understand the kind of functional difficulties that children will face, being the determinant for rehabilitation interventions.

Aside from this major evaluation, it is also important to evaluate the associated impairments that accompany the diagnosis of CP, such as sensation and perception deficits, and impairments in terms of cognitive, emotional, behavioral, communication, and social competences (Bax et al., 2005; Odding et al., 2006; Parkes et al., 2009). The consequences of these impairments extend to the Activities of Daily Life, with repercussions in the learning process (Mutsaarts et al., 2006; Van Rooijen et al., 2015). In fact, children with CP have a high risk of showing learning disabilities, which may arise before the schooling years (Jenks et al., 2009; Michel et al., 2011; Scope, 2013). The risk of learning disabilities is not determined exclusively by the cognitive impairment. In fact, children with CP with a normative cognitive level can still present specific learning difficulties (e.g., mathematics and reading; Frampton et al., 1998; Bottcher et al., 2009).

The specificities that characterize this population contribute to lower autonomy and participation in activities in their daily life. As previously mentioned, the indicators of CP picture contribute to the rising issue of a student’s feeling of alienation from school, which is at the root of low levels of SE.

### The Role of Parents in the Promotion of Autonomy and Participation

Parents can play a key role in the promotion of autonomy and participation of their children in daily life activities. Literature has shown that parental involvement in child development is a strong predictor of a positive educational trajectory (Barlow and Humphrey, 2012; Al-Alwan, 2014). The style of parental involvement can be a promoter of more, or less, autonomy and participation of children in their activities (Raftery et al., 2012). In fact, parenting behaviors have been classified in two dimensions: (i) parental Control and Restriction (CR), and Autonomy Allowance (AA); and (ii) parental Rejection and Hostility (RH), and Acceptance and Support (AS; Schaefer, 1965; Chorpita and Barlow, 1998; Aran et al., 2007). Regarding the first dimension, parental CR refers to overprotective behavior, excessive supervision, and imposition regarding the way children have to feel or think, or in the decisions they have to make; whereas, AA refers to the provision of opportunities to children to make their own decisions and be independent. The second dimension of parental behaviors integrates aspects of RH, which can be characterized by feelings of coldness and low desire to be, and interact, with the child. Finally, AS is characterized by positive emotional involvement with the child, implying active listening, care, and affection (Schaefer, 1965; Chorpita and Barlow, 1998; Aran et al., 2007).

Each style comprises elements of both dimensions; that is, the Autonomy Allowance and Acceptance and Support (AA/AS) parenting style, besides being a promoter of autonomy (Aran et al., 2007), is also a predictor of increased motivational strategies, with impacts on children’s achievement (Grolnick et al., 1997; Raftery et al., 2012). In fact, literature highlights that parental autonomy support promotes children’s self-regulation, motivation, and achievement and, consequently, their SE (Raftery et al., 2012). Conversely, the parental style of Control and Restriction and Rejection and Hostility (CR/RH) restrains the development of autonomous behaviors through restriction of opportunities and impediment on children to act freely (Aran et al., 2007). In fact, CR/RH emerges as the predominant parental style in parents/caregivers with children with disabilities, namely CP (Elad et al., 2013; Racine et al., 2013). For example, Elad et al. (2013) found that the mothers of children with hemiplegic CP scored their children performance lower than the therapists using clinical assessment protocols. This finding may be explained by the fact that mothers of children with disabilities are likely to perceive their children as vulnerable and low autonomous. This perceived lower autonomy often results in an overprotective set of behaviors, which could help explain why the CR/RH parental style is the most predominant among parents of children with CP. In sum, the parenting style can provide more, or less, orientation toward the learning process and may differently impact children SE.

### The Aim of This Study

Barlow and Humphrey (2012) stressed the need to analyze whether, and how, the parental styles described in literature are adopted by parents of children with Special Educational Needs and Disabilities (SEND). Findings are expected to help in the design of interventions well-fitted to educational practice and guidelines for educational policies. A recent meta-analysis (Castro et al., 2015) with typically developing students reports that parents who participate in, and closely follow, their children’s academic goals are likely to promote their SE. Additionally, findings from the same meta-analysis report that children less able to comply with the academic demands need more involved parents (Castro et al., 2015). Consistent with this proposition, authors (Castro et al., 2015) found that parental involvement in school activities and dynamics of children with SEND has positive outcomes in the promotion of SE. Still, despite the voluminous literature reporting positive relationships between parental involvement and SE, to our knowledge no study has yet examined how parents of children with CP perceive their involvement with their children’s school related activities and how this perceived parental involvement relates to the SE reported by their children.

Importantly, as Novak et al. (2013) highlighted, CP is considered the most common childhood physical disorder and researchers highlight the lack of investigation in the participation domain and the need of informing the literature by using qualitative and quantitative designs (Coster and Khetani, 2008; Kemps et al., 2011). Thus, we aim to understand the parental styles adopted by parents of children with CP in relation to their children’s level of autonomy and SE. To accomplish this aim, we explored the role of parental involvement in the promotion of autonomous behaviors in children with CP in relation to the SE reported by those children. Data collected from parents and students are expected to help researchers and educators in their work with children with CP.

## Materials and Methods

### Study Design

Two research questions guided our study: (1) How does the parental style promotes autonomous behaviors in children with CP?; (2) How does the parental style adopted by parents of children with CP relates to the level of SE reported by children?

To answer these questions, qualitative (i.e., interviews) and quantitative (i.e., questionnaire) approaches were followed. The parents of children with CP were interviewed about the daily and school routines of their children. The analysis targeted how parents perceived the autonomy of their children and how they promoted this autonomy, and provided indicators to define each parent/caregiver parental style. The children of the parents/caregivers interviewed were asked to fill in questionnaires assessing their SE.

The participation of parents and children was voluntary and unrewarded. Finally, informed consent were obtained from all the participants, being guaranteed data confidentiality. Additionally, all the participating parents/caregivers authorized the consultation of their children’s medical diagnoses to help researchers learn the children’s CP type and topographic classification of motor impairment. This study was carried out in accordance with the recommendations of the ethics committee of the University of Minho. All subjects gave written informed consent in accordance with the Declaration of Helsinki.

### Participants

This paper reports findings drawn from 19 parents/caregivers of children diagnosed with CP and from the 19 children of those parents. All participants attended CP rehabilitation centers in Portugal. In the first part of this study, the parents/caregivers were interviewed by two of the authors, trained to conduct semi-structured interviews. The second part involved the collection of SE questionnaires from the children with CP under the care of these parents/caregivers.

Regarding parents/caregivers, 17 are female and two are male, aged between 26 and 65 years (*M* = 42.22; *SD* = 8.83). Fifty percent of parents/caregivers completed the 9 years of the compulsory education, and the remaining graduated from high school, and from University (**Table 1**). Of the 19 children, 10 are female and nine are male, aged between 6 and 12 years (*M* = 9.89; *SD* = 2), and were in the elementary (*N* = 15, second to fifth grade) and in the middle school (*N* = 4, seventh grade). Participants attended three CP rehabilitation centers (approximate distribution among the three centers: 47, 42, and 11%). The majority of the children have the CP classification of hemiplegic and spastic (**Table 2**). All children, except one, attended mainstream schools. The other child attended special education classes in a mainstream school. Still, these classes are designed for children with SEND and the curriculum is adapted to each child’s needs.

**Table 1:** Demographic characteristics of participants: parents/caregivers (*N* = 19).

Participants characteristics – parents/caregivers of children with CP	
Age (years) mean ± SD	42.22 ± 8.83
**Gender *n* (%)**
Male	2 (10.5%)
Female	17 (89.5%)
**Degree of relatedness *n*(%)**
Mother	16 (84.2%)
Father	2 (10.5%)
Other Situation	1 (5.3%)
**Education level *n* (%)**
Elementary School	4 (21.1%)
Middle School	8 (42.1%)
High School	4 (21.1%)
Higher Education	3 (15.8%)

**Table 2:** Demographic characteristics of participants: children (*N* = 19).

Participants characteristics - children with CP	
Age (years) mean ± SD	9.89 ± 2
**Gender *n* (%)**
Male	9 (47.4%)
Female	10 (52.6 %)
**Type of CP *n* (%)**
Diplegia	7 (36.8%)
Right hemiplegia	7 (36.8%)
Left hemiplegia	2 (10.5%)
Without classification	3 (15.8%)
**Quality of tonus *n* (%)**
Spastic	14 (73.7%)
Diskenetic athetoid	2 (10.5%)
Ataxic	2 (10.5%)
Mixed	1 (5.3%)
**GMFCS *n* (%)**
Level I	11 (57.9%)
Level II	6 (31.6%)
Level III	2 (10.6%)

### Procedure

The Ethics Commission of University of Minho approved the study and all the interviewees authorized the recording of the interview using a digital recorder. A verbatim transcription was carried out to capture all the information. The interviews were conducted in person with a guarantee of privacy and confidentiality and lasted about 40 min each. Different researchers, all authors of this paper, were involved in the transcription and reviewing process. All data was stored in secured drives.

All the 18 Rehabilitation Centers in Portugal were contacted to participate in this study, five answered positively (a response rate of 28%). From these centers, three were randomly selected.

A total of 19 interviews of parents of children with CP and 19 questionnaires by their children were collected. The questions used in the interviews were selected from a semi-structured interview, Routine Based Interview (McWilliam et al., 2009), and focused on three dimensions: daily routines, school routines, and executive functioning. Parents were asked about their children’s daily and school routines, and to describe their involvement in those tasks (e.g., how you describe one normal day of your child?). Additionally, parents/caregivers describe their involvement in the promotion of their children’s autonomy and empowerment. Finally, parents/caregivers were inquired about the executive functioning of their children (e.g., Did he/she loose control frequently?). This last topic was not addressed in the present study.

The school engagement questionnaire used in the current study was adapted for the population of children with CP from the questionnaire by Wang and Holcombe (2010) validated to the adolescent normative population. In the current study, we used 13 items focusing on students’ perceptions of school engagement (e.g., For me is difficult to finish my homework; School is very important to me). One question from the original questionnaire was deleted because it did not match with this population (item 5: Getting a good education is the best way for me to get ahead in life in my neighborhood). Items were presented in a Likert-like format of five points (1 (never to 5 (always) and some of these items were in the positive format and others in the negative format. (χ^2^(366, *N* = 1046) = 1,105.36, *p* < 0.001; CFI = 0.92, TLI = 0.91, RMSEA = 0.05.

To complement these data, the medical and therapists records of the 19 children with CP were consulted regarding information about the type and topographic classification of the motor impairment, as well as on the GMFCS (Palisano et al., 1997; Andrada et al., 2012). This classification determines the level of motor function in different levels (I, minor difficulties to V, major difficulties). These indicators were collected to help frame each children autonomy pattern.

### Data Analysis

Interviews were analyzed using thematic analysis – by identifying and interpreting pattern themes (Braun and Clarke, 2006). To guide this process, outlined phases of thematic analysis described by Braun and Clarke (2006) were taken into account. Although a theory-based meaning was prioritized, codes emerged both in a “top down” and “bottom up” way. The codes allowed to find patterns, and connections between codes and to generate themes (Braun and Clarke, 2006). Before starting an in-depth analysis of the data, a coding frame (codebook) was developed based on the theoretical background. Subsequently, data were coded using a deductive approach in order to fit into these theoretical-driven codes. Yet, during coding, new codes emerged from the data (**Table 3**).

**Table 3:** Codebook of qualitative analysis.

Autonomy (Proot et al., 2000; Ryan and Deci, 2000; Cardol et al., 2002; Clapton and Kendall, 2002)	+ Autonomy	(i) Initiative; (ii) Make decisions; (iii) Capacity to perform (even when adapted to the limitations – physical or cognitive); (iv) Self-governance; (v) Self-awareness
	- Autonomy	
Parental Style (Schaefer, 1965; Chorpita and Barlow, 1998; Aran et al., 2007)	Parental autonomy allowance/acceptance and support	(i) Provide opportunities; (ii) Promotion of independence; (iii) Positive emotional involvement; (iv) Provide care.
	Parental control and restriction/ rejection and hostility	(i) Overprotective behavior; (ii) Excessive supervision; (iii) Control of feelings or thought? or decision-making.
School routines (Núñez et al., 2014, 2015a,b; Regueiro et al., 2015)	School activities	(i) Participation (how); (ii) School identification; (iii) Difficulties in school dynamics (e.g., physical barriers).
	Exam Preparation	(i) Initiative; (ii) Help requests; (iii) Schedule/Planning/Monitoring; (iv) Solving difficulties
	Homework task	(i) Initiative; (ii) Help requests; (iii) Schedule/Planning/Monitoring (iv) Solving difficulties.

To assist this qualitative data analysis, a set of tools from NVivo software was used. Created mainly as software to manage data, NVivo aims to facilitate the way to accede data efficiently (Bazeley and Jackson, 2013). Besides, NVivo queries (namely, coding queries and matrix coding queries) helped to check for patterns and to map interconnections between codes, allowing to gather them into emerging themes and to map connections between themes.

To ensure the precision of the coding scheme, inter-observer agreement was calculated. Two independent researchers with training on the coding scheme codified all the data. Raters discussed discrepancies in the coding scheme to reach a consensual coding. The second rater codified over 30% of the data and an almost perfect agreement was achieved (kappa coefficient = 0.89), according to Landis and Koch (1977). Main themes, subthemes, and interconnections between themes and subthemes were identified.

Regarding the questionnaire, for each child, scores of the individual items were summed and a total SE score was obtained. Thereafter, all total SE scores were ordered by quartiles. Finally, children in the first and second quartile were grouped in the Higher SE level, whereas children in the third and fourth quartile were grouped in the Lower SE level.

## Findings

**FIGURE 1:**
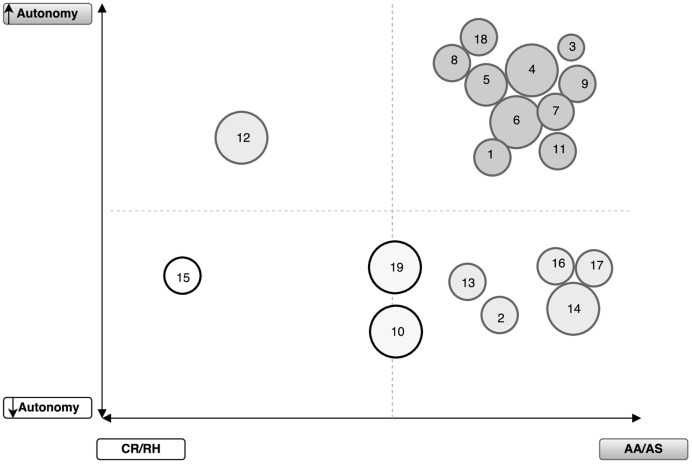
**Parental style and children autonomy reported by parents, contrasting with the level of motor functional impairment Gross Motor Function Classification System (GMFCS).** Each circle represents one child; the size represents the GMFCS level (large to level I – minor impairment; smaller to level III); the color of the circles represents the cross between the references of autonomy perceived and parental style. CR/RH, Control and Restriction/Rejection and Hostility; AA/AS, Autonomy Allowance/Acceptance and Support.

**FIGURE 2:**
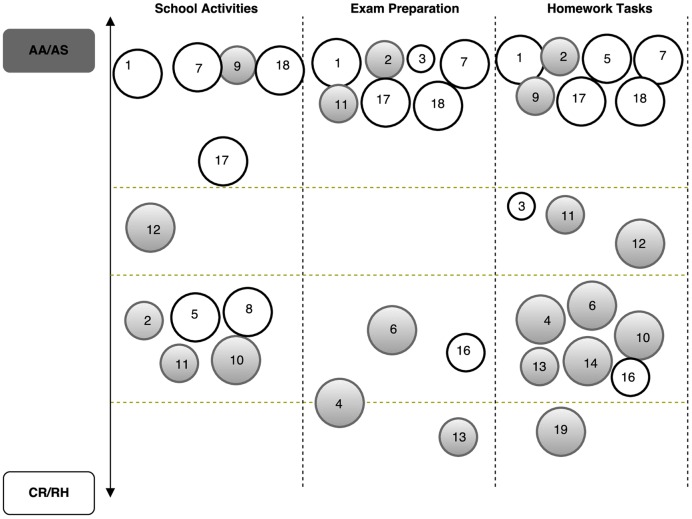
**Children’s school engagement (SE) and GMFCS level; and its relation with parental style.** Each circle represents one child; the size represents the GMFCS level (large to level I – minor impairment; smaller to level III); the gray circles represent higher SE level and white circles represents lower SE level. CR/RH, Control and Restriction/Rejection and Hostility; AA/AS, Autonomy Allowance/Acceptance and Support.

The data from the interviews were coded and themes were clustered into three main categories – autonomy level, parental style, and school routine (**Table 3**). To enhance comprehension, the analysis is presented in two different ways: (1) general analysis of the autonomy (regarding children daily routines) and parental style categories considering the GMFCS level of each child (**Figure 1**); and (2) case by node analysis regarding school routines, grouping results in three main categories (school activities, exam preparation, and homework tasks), considering the SE and GMFCS levels of each child (**Figure 2**).

Results are presented in **Figures 1** and **2**. In **Figure 1**, each circle represents a child and the size of the circle refers to the level of GMFCS (large size – level I and small size – level III). The color of the circles (one for each child) refers to children autonomy reported by parents (i.e., Percent of quotes from parents interviews stressing children autonomy) crossed with parental style (e.g., darker circles mean more perceived autonomy and more percent of quotes coded in the AA/AS parental style). In **Figure 2**, similarly to **Figure 1**, each circle represents a child, the size of the circle represents the level of GMFCS, and the color represents the level of SE (e.g., gray represents higher SE and white represents lower SE).

### General Analysis of the Perceived Children Autonomy and Parental Style

Parents were interviewed about the daily and school routines of their children and also about their involvement in those routines; data was used to draw the pattern of parental style. Besides, self-involvement reported by parents in those routines was taken into consideration to help classify children as more or less autonomous.

In general, respondents reported using strategies related to the parental style of AA/AS. Behaviors and strategies associated with this parental style are characterized by guiding children’s behavior to foster their self-involvement in daily routines.

Parent/caregiver 9 illustrates how parents promote autonomous behaviors through the strategy *training* during homework tasks:

Parent/caregiver 9: *When he returns home from school, he promptly does his homework. Now, we are training with him, and he does the homework alone… only asking for help to check whether the answers are right or wrong. If we don’t do that, he has the habit of asking for help all the time and needs to have one of us around him.*

Similarly, parent/caregiver 2 describes a situation at home in which the child helps in taking care of a little baby who stays during the workday with her parents, being the responsibility of this parent/caregiver:

Parent/caregiver 2: *I think the fact that she has a little [baby] at home, who we take care of, with us also helped her feel like her big sister. For a long time she asked us to give her the baby bottle and to change the diaper…. But… the diaper she’s not allowed, yet. Now, I think that the fact that the [baby] is with us at home is influencing her thoughts about the future. She asked me if she will be able to have a baby and to take care of her children. I answered: Yes, of course! You have to work hard. And I showed her success stories on the Internet…*

In this utterance, the parent/caregiver evaluates the level of autonomy and confidence of the child to help taking care of the baby. This allows the child feel a sense of autonomy and responsibility for her actions when taking care of someone else.

Regarding **Figure 1**, despite the GMFCS levels of the participating children ranging between levels I and III, participant parents/caregivers reported that their children display more autonomous behaviors than less autonomous. Nonetheless, the set of children for which parents/caregivers reported less autonomy behaviors mainly includes children with a clinical picture (e.g., mixed type, dyskinetic atethoid type) characterized by severe difficulties in performing some of the daily tasks mentioned in the interview (e.g., brush teeth, button up a coat).

The following situations illustrated by parent/caregivers 13 and 2 exemplifies the motor difficulties of their children and how they minimize their intervention while children execute the specific tasks (morning hygiene and dress by herself):

Parent/caregiver 13: *Her grandmother has to dress her… she goes to the bathroom, brushes her teeth, and brushes her hair, but all the things with her grandma’s help.*

Parent/caregiver 2: *She started to use a bra… and in this phase… she cannot dress by herself. She tries, but it gets stuck. She isn’t flexible enough and I end up doing it for her.*

#### Parental Autonomy Allowance/Acceptance and Support and Autonomous Behaviors

According to the literature, parents reporting AA/AS behaviors/strategies play an important role in the promotion of their children’s autonomy (Aran et al., 2007). Our data is consistent with the above; in fact parents/caregivers declare using more supportive behaviors with their children and, also, identify more autonomous behaviors in their children.

However, data also shows that the GMFCS levels were not related to the AA/AS behaviors/strategies parenting style and autonomy behaviors. In other words, higher motor functional impairment of the children did not show an impact in the autonomy perceived by their parents/caregivers.

Parents/caregiver 6: For example… when I go shopping in the supermarket, he sometimes goes with me and I ask: Please, go buy ham while I go get the fish. These little things I try to do…

Parent/caregiver 7: Now she knows that I only help her in the end to see if it is right or wrong [homework]. At the beginning I needed to be around her all the time.

Parents/caregivers reported to displaying AA/AS behaviors/strategies even when they perceive their children as less participatory in daily life activities. As mentioned above, less autonomous behaviors can be related to the constraints imposed by the clinical picture of the participants included in this cluster (**Figure 1**). Nonetheless, parents/caregivers utterances include references to strategies in the promotion of autonomy despite the specificities of the motor impairment. Parents/caregivers and children find strategies together to overcome the motor difficulties and be autonomous in daily activities (e.g., bath).

Parent/caregiver 2: *She sits and I put the clothes on her side and usually she dresses herself in the bathroom because its warm. Sometimes things may get complicated and she calls me: “Mother come here!” then I help her… only a little bit… I insist she do it by herself.*

Parent/caregiver 16: *She tends to call me because sometimes she did not understand the text and, ah, asks me if I can explain to her, if I can read… And I insist that she must try and complete the work by herself.*

#### Parental Control and Restriction/Rejection and Hostility and Autonomous Behaviors

Although less representative (only two cases), data shows that some parents/caregivers declared using the parental style of CR/RH to respond to more and less autonomous behaviors displayed by their children.

One of the children (child 12) is included in the cluster that refers to reported parental CR/RH behaviors and autonomous behaviors. This parent/caregiver mentioned using a parenting style with a control profile, despite the autonomous behavior exhibited by his child in most daily life activities.

It is important to stress that the AA/AS parental style is characterized by strategies such as active listening, care, and affection with the purpose of providing opportunities to the child be autonomous and feel a positive emotional involvement (Schaefer, 1965; Chorpita and Barlow, 1998; Aran et al., 2007). This parent/caregiver was able to recognize the motor competence of his child, but was not displaying the adequate strategies, as aforementioned, to help the child maintain these autonomous behaviors, as the following quotations illustrate:

Parent/caregiver 12: *Yes, she does everything… her part. She dresses herself. Once in a while I have to help to button up her coat… in the winter… She asks for help.*

Parent/caregiver 12: *For them [their children] to go, they need to go with me! Don’t get out alone. Is what I say if they want to go by themselves… if I don’t go, no one goes!*

Finally, the other parent/caregiver reported to displaying CR/RH behaviors and less autonomous behaviors when interacting with his child (15). Despite the low autonomy behaviors reported, child 15 is, still, close to the cutoff point for autonomous behavior (**Figure 1**).

Participant 15: *I always accustomed... I always accustomed [her] to do homework with the television on. If not, she doesn’t do anything.*

### Linking School Routines, SE, and GMFCS

As aforementioned, the results regarding the school routines, SE, and GMFCS will be interpreted as a function of the coded parental style (**Figure 2**).

Findings show that participants’ utterances were mainly related to an AA/AS parental style, regardless of the GMFCS and SE of their children. Within the autonomy AA/AS parental style, when compared to the other parental style, the majority of children reported a high level of SE. Even so, data displays a balance between the children who reported higher and lower SE levels.

Within the CR/RH parental style, the majority of children reported a high level of SE. Also, parents/caregivers who reported CR/RH parental style behaviors in the General Analysis of the Perceived Children Autonomy and Parental Style, maintained that style in relation to School Routines.

Findings also show that some parents/caregivers can range from an AA/AS parental style to a CR/RH parental style depending on the type of school activity under analysis (e.g., school routines, exam preparation, homework tasks; **Figure 2**). Moreover, irrespective of the SE level reported by children, some parents/caregivers reported CR/RH practices mostly related to Homework and Exam preparation tasks (e.g., 10, 13, 12, and 19).

Illustrative of low autonomy in the homework task, parent/caregiver 19 remarks that her presence is required for the child do homework. The parent is highlighting the need for a behavior change, such that the child becomes more autonomous and capable of performing the homework without her presence. However, there is no reference of strategies to change this behavior.

Parent/caregiver 19: *Sometimes… well, he only does the homework with me… Sometimes the dad is at home, but he doesn’t say that he has homework to do… Only when I arrive home, he says what he has to do.*

Regarding Exam Preparation, parents/caregivers 10 and 13 display, in their speeches, some signs of CR/RH behaviors with aspects related to the rejection/hostility dimension:

Parent/caregiver 10: *He doesn’t prepare for the exams. He only says to me ‘Tomorrow I will have an exam.’ And that’s it! He only brings the textbooks if he has homework to do. He never brings the books to study for an exam at home. Never...*

Parent/caregiver 13: *So, usually, as in this week, on Wednesday… I don’t know… I think it was on Friday; well never mind, she brought the books home and said for the first time that she had exams on Monday, Tuesday, and Wednesday…*

#### School Routines, Parental Style, and SE Level

The school routines category was subdivided into three subcategories: (i) School Activities; (ii) Exam Preparation; and (iii) Homework Tasks. The subcategory School Activities includes how children participate in school, the difficulties children face to cope with the school dynamics (e.g., the physical barriers to movement in the school building), and their sense of belonging. The Exam Preparation subcategory includes topics such as: the child’s initiative to self-set goals to the exam, study for exams, the monitoring strategies used while studying, the frequency of help requests (e.g., constantly or only when in doubt), and the child’s autonomy and strategies used to solve difficulties and problems. The Homework subcategory included the same topics with a focus on this specific task (e.g., initiative to self-set goals to do the homework).

The majority of the parents/caregivers are clustered in the AA/AS parental style, reporting displaying supportive practices to promote autonomous behaviors in relation to their children’s School routines; in particular, concerning homework and exam preparation assignments. Moreover, findings show a balance between children who reported high and low levels of SE.

Independent of the level of SE reported by children, parents/caregivers adopting an AA/AS parental style tend to promote their children’s autonomous behaviors, helping them deal with school responsibilities (e.g., be punctual to class, complete homework, bring the textbooks to class every day).

To clarify this finding, the following quotes represent two cases: AA/AS and higher SE (parent/caregiver 9), and AA/AS and lower SE (parent/caregiver 7):

Parent/caregiver 9: *Now we [parents] opt for asking to him to do the homework in the kitchen, because when we are cooking, he does his tasks. This way he can see us, feel safe, and do the work alone. He works independently, and only call us to see if the work is OK.*

Parent/caregiver 7: *Yes, yes. Now she no longer asks for my help all the time. She doesn’t call until the work is finished. In that moment I see if it is right or wrong...*

Findings also show that CR/RH practices, with greater focus on the rejection/hostility dimension, while less effective in the development of children’s autonomy, do not seem to be linked with lower levels of engagement behaviors in school tasks, as the following quotation suggests.

Parent/caregiver 5: *When he has exams… so we know about when he will have exams… and then we tell him ‘Hey [kiddo], don’t you have to study?’ ‘Ahhh, I studied, I know, it is not necessary, I have already studied…’*

## Discussion

The major goal of the present study was to understand the parental styles adopted by parents of children with CP in relation to their children’s levels of autonomy and SE. The majority of the parents/caregivers were clustered in an AA/AS parental style and the majority of their children were perceived as autonomous. Furthermore, neither children’s autonomous behaviors reported by parents, nor the parental style, seem to be related to the children’s SE or the GMFCS.

Moreover, findings showed that the AA/AS style encompasses both high and low levels of SE, and also that the CR/RH parental style is associated with higher levels of SE.

Furthermore, a main finding of the present study was that the majority of parents were identified as holding an AA/AS parenting style. This result is not consistent with the literature (Elad et al., 2013; Racine et al., 2013) and may be related to the fact that all the participating children attend rehabilitation centers in which a family centered approach is adopted. In the family centered approach the families are expected to be involved in the therapy of their children (e.g., physiotherapy). While participating in the sessions, parents/caregivers learn the appropriate strategies to adopt with their child to accomplish particular goals, being expected to reproduce them at home. Parents/caregivers attending rehabilitation centers with this framework are likely to be prone to understanding, in depth, the process followed by their children and to be prompt to develop strategies to support the autonomy of their child, because that is what they learn at the center. They are also prone to search for information and seek to understand the clinical needs of their children. The educational experience of these parents at the center is consistent with the AA/AS parental style and may help to explain findings. Moreover, these parents are likely to take a holistic approach to the health care, development, and learning of their child (Woodgate et al., 2015).

Despite the literature suggesting that children with disabilities, especially with physical disabilities, tend to show low autonomy in their daily life contexts (Simeonsson et al., 2001), still, in the current study, the majority of parents/caregivers did not describe their children’s behaviors as showing low autonomy (i.e., assessed by the level of GMFCS and by data extracted from the parents interviews). A possible explanation for this finding may be that parents of children with disabilities are more likely to value the little progresses accomplished by their children in terms of autonomy when compared with parents of children without disabilities (Blackorby and Cameto, 2004). Additionally, compared to professional evaluations, parents/caregivers tend to overvalue the level of functioning of their child (Keith and Markie, 1961).

Literature reports that the AA/AS parental style is related to the promotion of children’s autonomous behaviors (Aran et al., 2007). Still, interestingly, we found that a parent/caregiver identified as CR/RH (participant 12) perceived low autonomous behaviors in his child, but his child’s SE was high. Duncan and Caughy (2009) suggest a possible explanation for this mismatch. These authors mention that parents/caregivers of children with disabilities tend to perceive their children as vulnerable and in need of overprotection. Yet, children may not perceive their parents behavior as overprotective and, therefore, the parents’ behavior may not negatively influence the children’s SE level, as would be expected (Harper, 1977). Children with disabilities could perceive their parents/caregivers controlling behaviors as a pattern of security and comfort, with no influence on school perceptions and involvement (Eggland, 1973; Cohen et al., 2008).

Our findings suggest that parental style is an important variable with impact on children’s SE, but also stress the need to consider other variables that may influence the level of SE, such as the way children perceive school or their future academic expectations (Cohen et al., 2008). Researchers might consider examining parents parenting style perceived by children with CP and the motives for displaying particular educational strategies (e.g., instigate autonomy by pushing children to dress themselves). Findings are expected to help researchers understand the complex relationships between parenting styles, the promotion of autonomy, and SE. For example, to understand why almost half of the children in our study reported higher SE, and the other lower, despite the parents/caregivers displaying an AA/AS parental style. Additionally, researchers could also consider analyzing data beyond the parent-child dyad. For example, understand how the systems and structures that surround the child may promote or restrict the child’s autonomous behavior.

This finding may be explained because children, despite their parents’ efforts and involvement in school activities, anticipate their future after school as difficult and without opportunities for people with their clinical condition (Connell, 1990; Skinner and Belmont, 1993). Another hypothesis to clarify this finding may be that parents/caregivers perceiving their child as not being able to be successful in school or in a professional course are likely to devalue schoolwork and show low involvement in school activities (e.g., helping with homework). Rather, parents/caregivers focus their children educational goals on areas of functioning (e.g., physical recuperation) other than school. Children with parents holding these beliefs may face difficulties in their commitment to school and show a high SE, but still they can achieve school success.

Our results reveal other aspects of the complex nature of the parenting styles construct. Parents/caregivers with a particular parenting style were not consistent with that style with all the different activities analyzed. For example, some parents/caregivers were identified as using the AA/AS parenting style in some school routines (e.g., homework tasks) and the CR/RH parenting style in others (e.g., school activities and exam preparation). This finding stresses the need to analyze the parenting style in relation to particular tasks. Homework, for example, being an universal instructional strategy (Núñez et al., 2015b; Valle et al., 2016), is likely to be conceptualized by parents as a promoter of academic learning, which could lead parents to be prone to encourage their children’s autonomy in this activity (Cunha et al., 2015).

Parents/caregivers lacking consistency in their parenting styles may have difficulty in promoting their children’s autonomy in all contexts of daily life. Still, in the current research, some children with parents holding inconsistent parenting styles reported high SE.

### Limitations and Future Studies

Findings on children’s autonomous behaviors reported by parents and the parental style provide a corpus of knowledge that is expected to help in the design of interventions fitted to particular family needs. Still, despite being promising, our results are preliminary, present limitations, and should be further investigated. First, the study is focused on children’s autonomous behaviors reported by parents, as well as on parental involvement in their children’s daily life routines. So, data cannot be generalized, but only understood in light of the study’s participants. Qualitative research does not intend to generalize findings to a larger population, but to facilitate the transferability of the results, for example informing and facilitating insights into contexts other than that in which the survey was conducted (Carpenter and Suto, 2008). Second, participants (i.e., parents/caregivers and their children) attend rehabilitation centers following an approach focused on family collaboration, which could limit the phenomenon comprehension. Further studies with children from rehabilitation centers following different types of theoretical frameworks, or with children who do not attend any rehabilitation center, are needed to compare findings. Third, SE was assessed with a self-report measure. Future research might consider including other ways to assess this construct, such as class observation or self-reports using other sources of information. Lastly, we did not include parents’ parental style perceived by children. This measure could have increased the trustworthiness of the findings. To address these limitations, researchers might consider, for example, crossing the perceived autonomy of children with CP and SE with the children’s autonomy and SE perceived by parents.

This information would help to disclose the complex relationships between parental involvement and children outputs, and to design tailored interventions fitted to the educational needs of children with CP. Moreover, Aran et al. (2007) found that the parental style has more impact in the quality of life of children with CP than in their siblings’ quality of life (typically developed). Therefore, it would be interesting to analyze if the impact of the parental style in the specific case of children with CP is different in the case of typically developing children.

Rehabilitation centers, and also schools, could consider organizing training addressing parents/caregivers’ educational needs. A close relationship between the strategies used by therapists while working with children with CP (e.g., physiotherapists) and those used by parents/caregivers at home is expected to help children become more autonomous (Pereira et al., in press). Additionally, this training could promote parents/caregivers reflections on parental styles and SE, stress the practical applicability of promoting these strategies with their child, and foster their involvement.

## Author Contributions

AP, TM, SL, and AN were responsible for the literature search, data collection, data analysis, and data interpretation. PM, SF, and NR were in charge of technical guidance. PR and JN made important intellectual contribution in research design and manuscript revision. All authors were involved in the writing process of this manuscript.

## Conflict of Interest Statement

The authors declare that the research was conducted in the absence of any commercial or financial relationships that could be construed as a potential conflict of interest.
